# Theories on the Link Between Autism Spectrum Conditions and Trans Gender Modality: a Systematic Review

**DOI:** 10.1007/s40489-022-00338-2

**Published:** 2022-08-19

**Authors:** Luna L. Wattel, Reubs J Walsh, Lydia Krabbendam

**Affiliations:** 1 Faculty of Science, University of Amsterdam, Science Park 904, 1098 XH Amsterdam, Netherlands; 2 Department of Clinical, Neuro- and Developmental Psychology, Faculty of Behavioural and Movement Sciences, Vrije Universiteit, Van der Boechorststraat 9, 1081 BT Amsterdam, Netherlands

**Keywords:** Autism spectrum conditions, Autism spectrum disorder, Trans gender modality, Gender dysphoria, Co-occurrence

## Abstract

While research on the prevalence of co-occurring autism spectrum conditions (ASC) and trans gender modality (TGM) is available, less is known about the underlying mechanism of this association. Insight is needed to improve treatment of trans autistic people. This review provides an overview of theories on the ASC-TGM link and the available evidence for/against them published between January 2016 and October 2020. A systematic search was performed in PubMed, PsycINFO, Web of Science, and Scopus. This resulted in 36 studies, in which 15 theories were identified. Results indicate all theories lack substantial empirical support. Unlikely and promising theories were identified. The most promising theories were those on resistance to social norms and weakened sex differences. Future directions are provided.

## Introduction

According to the Diagnostic and Statistical Manual of Mental Disorders (DSM-5), autism spectrum disorder (ASD) is a neurodevelopmental disorder characterized by impaired social communication and social interaction in combination with restricted interests and repetitive behaviors (American Psychiatric Association, APA, 2013). This definition of autism has been criticized, however. According to the neurodiversity movement, autism is better understood in terms of natural variation than in terms of deficits (Rowland, 2020; Jaarsma & Welin, 2012). The neurodiversity model focuses on autism as an identity or a dimension of individual difference, while the deficit model of autism (as implied by the term ASD) focuses on causation and cure (Kapp et al., 2013). Therefore, the term autism spectrum conditions (ASC) will be used in this review. The prevalence estimates for ASC have changed over the last decades, with estimates in American children increasing from 1:150 to 1:54 between 2000 and 2016 (Chiarotti & Venerosi, 2020). It remains difficult to distil whether these numbers reflect an actual increase in ASC or rather a change in diagnostic practices and awareness (Liu et al., 2010). Males are roughly four times more likely to be diagnosed with ASC than females (Loomes et al., 2017; Maenner et al., 2020). In terms of etiology, interactions of genetic and epigenetic factors have been found to play a significant role in the development of ASC (Yoon et al., 2020). Still, some of these genetic and nongenetic predispositions remain uncertain (Myers et al., 2020).

Gender modality is a term that describes how a person’s gender identity stands in relation to the gender assigned at birth (Ashley, 2021). A person’s gender modality may be cisgender (or cis), transgender (or trans), or any other modality (e.g., agender). In this paper, the term trans gender modality (TGM) will be used to refer to all modalities in which the person’s gender identity is different from their birth-assigned gender. This experienced difference may lead to gender dysphoria. Gender dysphoria (GD) is a diagnosis specified in the DSM-5 that has replaced previous diagnoses like gender identity disorder and transsexualism (APA, 2013). Although both TGM and GD refer to an incongruence between experienced and birth-assigned gender, there is a difference. While TGM is typically permanent, GD requires impaired functioning or distress, which is generally resolved through social and/or medical transition (APA, 2013; van de Grift et al., 2017). The prevalence of GD has been estimated between 4.6 and 6.8 per 100,000 individuals (Arcelus et al., 2015; Collin et al., 2016). Similar to the growth in ASC prevalence, an increase in referrals for GD has been reported in northern Europe (Kaltiala et al., 2020). Again, it is difficult to distinguish a cause of this apparent growth other than the effect of changed diagnostics and societal acceptance (Kaltiala et al., 2020). Several environmental and biological hypotheses on the etiology of TGM have been proposed throughout history (Ettner, 2020), but many of these hypotheses have been challenged (cf. Lair 2016; Auer et al., 2016; Fuss et al., 2013).

The interest in the co-occurrence of ASC and TGM has grown. In just a few years, the number of articles published on the topic has tripled: where reviews written in 2016 contained circa 10 original data studies (Glidden et al., 2016; van der Miesen et al., 2016), a recent review contained around 30 (Thrower et al., 2020). In one of the first reviews on the ASC-TGM link, Glidden et al. (2016) conclude an overall high prevalence of ASC in trans people. However, they emphasized that the included literature was limited—especially for adults (i.e., three quantitative studies). Although Glidden et al. (2016) discussed some potential explanations for the co-occurrence of ASC and TGM, van der Miesen et al. (2016) have covered this topic more thoroughly. They discussed the underlying hypotheses extensively in three categories: biological, psychological, and social explanations. Van der Miesen et al. (2016) note that all the discussed hypotheses lack evidence. Similar to Glidden et al. (2016), van der Miesen et al. (2016) conclude there is evidence for a link between ASC and TGM despite the limited literature. The most recent publication on the matter is a systematic review by Thrower et al. (2020), which focused on the prevalence of ASC and attention-deficit/hyperactivity disorder in trans people. They included 21 papers on ASC in trans people and eight papers on TGM in autistic people. Thrower et al. (2020) conclude that their findings suggest a prevalence of ASC in 6–26% of trans people. This is significantly higher than the prevalence of ASC in the general population, which has been estimated to be around 1.85% (Maenner et al., 2020). In short, all three discussed reviews demonstrate an increasingly established link between ASC and TGM in a fast-growing body of literature.

Instead of focusing on the question “is there a link?” (see Glidden et al., 2016; Thrower et al., 2020), the current review will focus on the question “why is there a link?.” The aim of this paper is to provide an update of (a) the existing theories on the relationship between ASC and TGM; (b) the available evidence for or against those theories. Since 2016, no structured report of the underlying hypotheses on the co-occurrence of ASC and TGM has been provided. Therefore, this review will build on the work of van der Miesen et al. (2016) by analyzing the available literature from 2016 onward.

It is important to gain insight into the underlying mechanisms that contribute to the co-occurrence of ASC and TGM in order to provide proper treatment. Clinicians specialized in TGM are not generally trained in working with autistic people and vice versa for clinicians specialized in ASC. Although some initial guidelines for adolescents with co-occurring ASC and TGM have been proposed (Strang et al., 2018a), complete and evidence-based insight is lacking. By providing an overview of the available theories on the ASC-TGM link since 2016, this review will further the understanding of the relationship between ASC and TGM. With regard to treatment and awareness, this could prove relevant for scientists, clinicians, trans autistic people, and the public alike.

For comprehensibility, the review is structured across two dimensions. First, an overview according to study type is provided (i.e., quantitative and qualitative studies). Second, the literature is reviewed per identified theory on the ASC-TGM link. In the discussion, the strengths and weaknesses of the support provided for or against each theory are evaluated and future directions are provided.

## Methods

### Literature Search

The literature search was conducted between October and November of 2020. Four different databases were searched from 2016 onward: PubMed, PsycINFO, Web of Science, and Scopus. Literature was searched for ASC terms (autism, autism spectrum disorder, autis*, and asperger*) and TGM terms (gender dysphoria, gender identity disorder, transgender*, and transsex*). Search terms were combined using Boolean operators “AND” and “OR.”

### Eligibility Criteria

Of interest were studies containing empirical data and theories on the link between ASC and TGM. Theories were defined as theoretical (i.e., not methodological) explanations given for the ASC-TGM link. Studies without empirical data or without theories were excluded (e.g., reviews, letters, and opinion articles). Articles were considered eligible if they were peer-reviewed, published in English, and available in full text.

### Study Selection

First, duplicates were removed (*n* = 21). Next, irrelevant articles were removed in three stages: based on title (*n* = 111); based on abstract (*n* = 55); based on full text (*n* = 13). Three articles were removed because there was no peer-reviewed, full-text English publication available. The removal of duplicates and the removal based on title and abstract were performed by the first author. The removal based on the full text was performed by the first and second authors in consultation. In total, 36 articles were included. The included articles are marked with an asterisk in the reference list.

### Data Items

For each included article, the following data were recorded: participant characteristics (birth-assigned sex, age, and diagnosis/gender modality); study characteristics (study type, measurements, and sample size); outcomes (relevant results, mentioned theories, and claimed support for/against theories). The latter two outcomes are specified below.

### Theory Identification and Classification

The outcome “mentioned theories” was defined as any theory being named in the text, regardless of the authors’ data or arguments. The outcome “claimed support for/against theories” was defined as any theory that was argued for or against by the authors based on their own data. Theories that were argued for or against but without reference to the authors’ own data were categorized as mentioned theories. Identification of the theory categories was performed by the first author. Classification of the theories into categories was performed independently by the first and second authors. Where classification between both did not correspond, the authors consulted and reached an agreement.

## Results

### Quantitative Literature

A total of 30 studies containing quantitative data were included: nine on ASC populations, 16 on TGM populations, and five on the general population. Of the 30 papers, 17 included a comparison to control groups or normative data, and two contained longitudinal data. Table 1 provides an overview of all quantitative papers organized by study population and age cohort.

**Table 1:** Overview of the quantitative literature ordered by study population

Authors (year)	Study type	Sample (AMAB)	Relevant findings	Mentioned theories	Claimed evidence for/against
ASC population: children and adolescents					
Hisle-Gorman et al. (2019)	Chart review	48.762 (39.010)	Elevated rates of GD diagnoses (ICD-9) in autistic children (0.07%) compared to matched controls (0.01%)	Gender development Genetic factors	-
Janssen et al. (2016)	Chart review	492 (409)	Elevated rates of TGM (CBCL item 110) in autistic children/adolescents (5.1%) compared to controls (0.7%)	Birthweight Resistance to social norms	-
May et al. (2017)	Cross-sectional study	176 (136)	TGM (CBCL item 110) in autistic children/adolescents (4.0%) elevated compared to non-referred controls (0.7%) and similar to referred controls (4.0%)	Gender development Obsessions Prenatal hormones Resistance to social norms ToM/mentalizing Weakened sex differences	-
ASC population: adolescents and adults					
Cooper et al. (2018)	Cross-sectional study	219 (118)	Elevated rates of TGM (multiple-choice question) in autistic adults compared to controls. Lower levels of gender identification and gender self-esteem (custom questionnaires) in autistic adults compared to controls	Feeling different Gender development Minority stress Prenatal hormones Resistance to social norms Social communication	-
Dewinter et al. (2017)	Cross-sectional study	675 (326)	TGM (multiple-choice question) in 8% of autistic AMABs and in 22% of autistic AFABs	Feeling different Prenatal hormones Resistance to social norms	-
George and Stokes (2018)	Cross-sectional study	310 (90)	Elevated rates of TGM (multiple-choice question) in autistic adults (AMAB: 22.2% and AFAB: 32.9%) compared to controls (AMAB: 6.9% and AFAB: 12.7%). Elevated GD traits (GIDYQ) in autistic adults compared to controls. GD traits partially mediated the relationship of ASC traits to sexual orientation	Extreme male brain Obsessions Prenatal hormones Resistance to social norms Rigidity Sexual orientation Social communication	-
Pecora et al. (2020)	Cross-sectional study	134 (0)	Elevated rates of TGM (multiple-choice question) in autistic AFABs (19.4%) compared to neurotypical AFABs (8.7%)	Obsessions Prenatal hormones Resistance to social norms Rigidity	-
Walsh et al. (2018)	Cross-sectional study	669 (322)	TGM (multiple-choice question) in 15% of autistic adults. Elevated cognitive autism traits (AQ-Short) and lower visual and auditory hypersensitivity (SPQ) in trans autistic adults compared to cis autistic adults	Obsessions Resistance to social norms Rigidity	Against obsessions Against rigidity For resistance to social norm
van der Miesen et al. (2018b)	Cross-sectional study	573 (469) adolescents 807 (616) adults	Elevated rates of TGM in autistic adolescents (YSR; 6.5%) and autistic adults (ASR; 11.4%) compared to controls (3.1% and 5.0% respectively). No significant associations between TGM (YSR/ASR) and specific subdomains of ASC (CSBQ/ASBQ) in adolescents or adults	Extreme male brain Feeling different Minority stress Obsessions Prenatal hormones Resistance to social norms Rigidity ToM/mentalizing Weakened sex differences	Against obsessions Against rigidity
TGM population: children and adolescents					
Akgül et al. (2018)	Cross-sectional study	25 (13)	Elevated rates of clinical range scores on SRS in trans children/adolescents (68%) compared to cis controls (22%)	Feeling different Prenatal hormones Social communication	-
Hill et al. (2020)	Chart review	13 (0)	Similar MACI scores between trans adolescent AFABs and cis adolescent AFABs admitted to a secure forensic adolescent hospital	Obsessions Rigidity Social communication	-
Holt et al. (2016)	Chart review	218 (81)	ASC (chart diagnosis) in 13.3% of GD-referred youth	Rigidity	-
Leef et al. (2019)	Chart review and cross-sectional study	61 (45)	Elevated rates of ASC (chart diagnosis) in GD-referred children (21.3%) compared to referred controls (0%). Similar SRS scores between GD-referred children and referred controls	Minority stress Obsessions Rigidity	-
Russell et al. (2020)	Longitudinal study	95 (38)	Similar SRS-2 score before and after treatment in GD-referred adolescents	Minority stress	-
Shumer et al. (2016)	Chart review	39 (22)	Possible ASC (ASDS) in 23.1% of GD-referred youth	Genetic factors Prenatal hormones Resistance to social norms Rigidity	-
van der Miesen et al. (2018a)	Cross-sectional study	490 (248)	Elevated rates of possible ASC (CSBQ) in GD-referred children (14.5%) compared to controls (3.5%). Similar CSBQ scores between AMABs and AFABs	Birthweight Extreme male brain Feeling different Minority stress Obsessions Prenatal hormones Rigidity Sensory processing Social communication	Against extreme male brain Against rigidity Against obsessions For sensory processing For social communication
Zucker et al. (2017)	Validation cross-sectional study	386 (304)	Elevated rates of obsessions (TRF items 9) in GD-referred AMABs (41.7%) and GD-referred AFABs (38.0%) compared to referred and non-referred controls	Birthweight Genetic factors Obsessions Prenatal hormones	For obsessions
TGM population: adults					
Cheung et al. (2018)	Chart review	540 (n.a.)	ASC (chart diagnosis) in 4.8% of trans adults	Genetic factors Minority stress Prenatal hormones	-
Fielding and Bass (2018)	Chart review	153 (97)	ASC (chart diagnosis) in 7.8% of GD-referred adults	Feeling different Rigidity	-
Heylens et al. (2018)	Chart review Cross-sectional study	532 (351) 63 (33)	ASC (chart diagnosis) in 6.02% of GD-referred adults. Possible ASC (AQ) in 4.84% of GD-referred adults. Elevated rates of clinical range on SRS in GD-referred adults (27.11%) compared to controls. SRS scores did not significantly differ between AMABs and AFABs	Extreme Male Brain Minority stress Prenatal hormones Social communication	Against Extreme Male Brain
Kung (2020)	Cross-sectional study	323 (145)	Possible ASC (AQ) in 11.0% of trans adults. Elevated ASC traits (AQ, SQ-S, EQ-S, EQ-10, RMIE) in trans AFABs compared to normative data. Similar ASC traits in trans AMABs and normative data	Extreme Male Brain Prenatal hormones Rigidity ToM/mentalizing	For Extreme Male Brain For ToM/mentalizing
Nobili et al. (2018)	Cross-sectional study	656 (396)	Elevated rates of possible ASC (AQ-Short) in trans AFABs (45.4%) compared to cis AFABs (30%). No significant difference in rates of possible ASC between trans and cis AMABs. Elevated rates of possible ASC in trans AFABs (45.4%) compared to trans AMABs (30.3%)	Extreme Male Brain Minority stress	For Extreme Male Brain
Nobili et al. (2020)	Longitudinal study	118 (59)	Similar rates of possible ASC (AQ-Short) before (34.7%) and after (32.2%) gender-affirming treatment in trans adults. Higher overall AQ-Short scores in AFABs compared to AMABs	Extreme Male Brain Minority stress Prenatal hormones	For Extreme Male Brain Against minority stress
Stagg & Vincent (2019)	Cross-sectional study	177 (66)	Elevated rates of ASC (self-reported diagnosis) in trans adults (14%) compared to cis adults (4%). Elevated autistic traits (AG, EQ, SQ, RMIE) in trans adults compared to cis adults, driven by AFABs	Extreme male brain Feeling different Minority stress	For Extreme Male Brain Against Extreme Male Brain
Vermaat et al. (2018)	Cross-sectional study	326 (191)	Similar rates of possible ASC (AQ) in GD-referred adults (9.5%) and controls (8.0%). Positive association between ASC traits (AQ) and GD intensity (UGDS)	Extreme Male Brain Minority stress Rigidity Sexual orientation Weakened sex differences	For weakened sex differences
General population: children					
Nabbijohn et al. (2019)	Cross-sectional study	2.445 (1.247)	Association between TGM (GIQC) and specific ASC characteristics (CSBQ) in nonclinical children. Association between TGM (GIQC) and parent-reported diagnoses of ASC, SPD and ODD in children with developmental/mental health diagnoses	Birthweight Extreme male brain Gender development Minority stress Obsessions Prenatal hormones Resistance to social norms Sensory processing Social communication	For sensory processing Against social communication
General population: adults					
Kallitsounaki and Williams (2020b)	Cross-sectional study	101 (51)	Association between ASC traits (AQ) and recalled and current GD feelings (RCGI and GIDYQ) in adults. Mentalizing (RMIE) moderated the relation between ASC traits and current GD feelings	ToM/mentalizing Resistance to social norms	For ToM/mentalizing (moderating role)
Kallitsounaki and Williams (2020a)	Cross-sectional study	101 (51)	Negative association between ASC traits (AQ) and explicit gender self-concept (PAQ) in adults. Negative association between ASC traits and implicit gender self-concept (IAT)	Gender development ToM/mentalizing Weakened sex differences Resistance to social norms	For weakened sex differences
Kallitsounaki et al. (2020)	Cross-sectional study	126 (29)	Replication of Kallitsounaki & Williams (2020b). Mentalizing mediated the relation between ASC traits (AQ) and current GD feelings (GIDYQ)	Gender development ToM/mentalizing Resistance to social norms	For ToM/mentalizing (moderating role)
Warrier et al. (2020)	Cross-sectional study	641.860 (n.a.)	Higher rates of ASC in trans adults compared to cis adults. Higher scores on self-report measures of ASC traits (AQ-10, EQ-10, SQ-10, and SPQ-10) in trans adults compared to cis adults	Feeling different Minority stress Prenatal hormones Resistance to social norms	-

Abbreviations: *TGM*, trans gender modality; *GD*, gender dysphoria diagnosis; *ASC*, autism spectrum conditions; *AMAB*, assigned male at birth; *AFAB*, assigned female at birth; *SPD*, sensory processing disorder; *ODD*, oppositional defiant disorder; *ToM*, theory of mind; *SRS*, social responsiveness scale; *MACI*, Millon Adolescent Clinical Inventory; *ASDS*, Asperger Syndrome Diagnostic Scale; *C/ASBQ*, Children’s/Adult Social Behavior Questionnaire; *TRF*, Teacher’s Report Form; *AQ*, autism spectrum quotient; *SQ-S*, systemizing quotient-short; *EQ-S*, empathy quotient-short; *RMIE*, Reading the Mind in the Eyes Test; *UGDS*, Utrecht Gender Dysphoria Scale; *CBCL*, Child Behavior Checklist; *GIDYQ*, Gender-Identity/Gender-Dysphoria Questionnaire for Adolescents and Adult; *ICD-9*, International Statistical Classification of Diseases and Related Health Problems, Ninth Revision; *SPQ*, sensory perception quotient; *Y/ASR*, Youth/Adult Self Report; *GIQC*, Gender Identity Questionnaire for Children; *RCGI*, Recalled Childhood Gender Identity/Gender Role Questionnaire; *PAQ*, Personal Attributes Questionnaire; *IAT*, Implicit Association Test

### Qualitative Literature

A total of six studies with qualitative data were included. Of those, four interviewed trans autistic people, one interviewed the mothers of gender-diverse children, and one interviewed clinical experts. Table 2 provides an overview of the qualitative data.

**Table 2:** Overview of the qualitative literature

Authors (year)	Case (gender)	Short description	Mentioned theories	Claimed evidence for/against
Cain and Velasco (2020)	1 autistic adult (AFAB nonbinary)	The participant explains their ASC makes it difficult for them to conceptualize gender as something that relates to them. They mention that they think a lot of autistic people have a propensity to be trans, but are not sure about the reason, especially about it being social (i.e., related to interacting with other people). The participant talks about feeling left out in childhood and shares their struggles with being “different.”	Feeling different Minority stress Resistance to social norms Social communication	-
Coleman-Smith et al. (2020)	10 autistic adults (5 trans men, 3 trans women, 1 nonbinary and 1 queer)	Some participants said that social communication difficulties and a different gender experience were a “double whammy,” mentioning feeling different and being bullied. Other participants described ASC as a protector against fear of negative perceptions. The authors state that ASC may pose a barrier to interpersonal gender exploration. One participant explained how ASC increased her gender dysphoric distress (e.g., the EMB theory implying she has a male brain). Another participant described how focusing on transgenderism might have been a ‘singular focus’ cognitive bias due to their ASC	Feeling different Extreme male brain Minority stress Obsessions Resistance to social norms Social communication	-
Hillier et al. (2019)	4 autistic adults (1 transgender male, 2 agender and 1 queer)	One participant said that ASC had not affected their understanding of gender identity, but their gender identity itself. They elaborate saying they feel like having ASC made them live more in their mind, separated from a connection with their body. The authors conclude that holding multiple minority identities aggravates social challenges	Gender development Minority stress	-
Kuvalanka et al. (2018)	3 autistic children (1 AFAB and 2 AMABs)	Mothers of three gender-diverse autistic children were interviewed. One child identified as a “boy-girl” at age 9 but again as a boy at age 12. His mother said his ‘obsession’ with gender had passed. Two mothers felt their child’s social problems/anxiety likely stemmed from ASC but wondered if gender nonconformity also played a role	Minority stress Obsessions	-
Strang et al. (2018a)	22 clinicians	Expert clinicians did not reach consensus on the presence of overfocused gender-related interests in autistic adolescents. A majority noted that gay/lesbian autistic adolescents sometimes assumed that they are a different gender because of their sexual attraction, although several clinicians reported never experiencing this. According to the authors, diagnosing ASC in trans youth is complex because they might appear socially awkward or withdrawn due to their TGM. The authors state that autistic adolescents may have a ‘black-and-white’ idea about gender. The authors additionally note that some autistic children/adolescents are unconcerned with or unaware of social expectations when coming out	Minority stress Obsessions Resistance to social norms Rigidity Sexual orientation	-
Strang et al. (2018b)	22 autistic adolescents (6 trans men, 14 trans women, and 2 nonbinary)	Some participants showed little interest in traditional gender presentations. The authors deduce that a lessened pressure to conform to gender stereotypes might be due to ASC. Many participants described gender exploration as a key experience to learn about their gender. The authors speculate that gender exploration is a primarily abstract thought process, posing challenges to autistic people. The authors conclude that their findings oppose the idea that TGM in ASC are driven by superficial “obsessional” interests	Obsessions Resistance to social norms ToM/mentalizing	Against obsessions

Abbreviations: *TGM*, trans gender modality; *ASC*, autism spectrum conditions; *AMAB*, assigned male assigned at birth; *AFAB*, assigned female assigned at birth; *EMB*, extreme male brain

### Theories

**Fig. 1:**
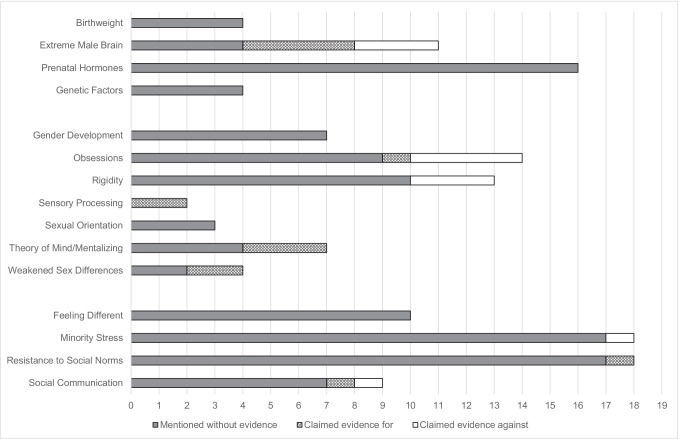
Number of mentions and claims of evidence for/against per theory on the ASC-TGM link throughout the 36 included papers

Building on the eight theories itemized in van der Miesen et al. (2016), 15 theories were identified. Table 3 offers a short description of each theory. The number of mentions per theory and the number of claimed evidence for or against each theory are also included in Table 3. Figure 1 illustrates the number of mentions and claimed evidence for/against per theory. Each theory is explained in detail and illustrated by means of the included literature below.

**Table 3:** Overview of theory types with the frequency of mentions and empirical evidence for or against

Theory type	Short description	Implied mechanism	Mentions	Claimed evidence for	Claimed evidence against
Biological explanations			35	4	3
Birthweight	High birthweight is a biological marker for both ASC and TGM	Common factor	4	-	-
Extreme male brain	An extremely male brain is associated with both ASC and TGM (specifically in AFABs)	Common cause	11	4	3
Prenatal hormones	Prenatal androgen exposures contribute to the development of ASC and TGM	Common cause	16	-	-
Genetic factors	There is a common underlying genetic predisposition for ASC and TGM	Common cause	4	-	-
Psychological explanations			50	8	7
Gender development	Atypical gender development in autistic people leads to high prevalence of TGM	ASC → TGM	7	-	-
Obsessions	Obsessions are a manifestation of ASC leading to (temporary) TGM Obsessions are an expression of TGM leading to specific ASC traits (i.e., restricted interests)	ASC → TGM TGM → ASC	14	1	4
Rigidity	Cognitive inflexibility due to ASC causes more rigid and concrete thinking about gender, making autistic people more prone to TGM	ASC → TGM	13	-	3
Sensory processing	Over- or under-responsivity to specific sensory input is a common factor in ASC and TGM	Common factor	2	2	-
Sexual orientation	ASC leads to a stronger relationship between sexual orientation and gender experience. Autistic people may assume to be of another gender when sexually attracted to the same gender	ASC → TGM	3	-	-
ToM/mentalizing	Deficits in ToM/mentalizing abilities in autistic people lead to TGM	ASC → TGM	7	3	-
Weakened sex differences	TGM in autistic people is due to weakened sex differences (less femininity/masculinity in autistic people)	ASC → TGM	4	2	-
Social explanations			55	2	2
Feeling different	TGM in autistic people is associated with feeling different from others in their assigned gender group ASC traits in trans people reflect feeling different due to TGM, not actual ASC	ASC → TGM TGM → ASC	10	-	-
Minority stress	ASC traits in trans people reflect social difficulties due to TGM, not actual ASC Minority stress is a shared factor in the ASC-TGM link, as both are marginalized groups	TGM → ASC Common factor	18	-	1
Resistance to social Norms	TGM is more common among autistic people compared to neurotypical people because they are less susceptible to social norms/prejudice	ASC → TGM	18	1	-
Social communication	Deficits in social communication due to ASC result in little understanding of gender norms, making autistic people more prone to TGM Problems with social communication are a shared phenomenon between ASC and TGM	ASC → TGM Common factor	9	1	1

Abbreviations: *TGM*, trans gender modality; *ASC*, autism spectrum conditions; *AFAB*, assigned female at birth; *ToM*, theory of mind

### Biological Theories

#### Birthweight

According to this theory, the common factor between ASC and TGM is high birth weight. Although the mechanism through which high birthweight would lead to either ASC or TGM remains unclear, it was proposed as a biological marker for both. There were four mentions of this theory and no claims of evidence for or against it. All four papers (Janssen et al., 2016; Nabbijohn et al., 2019; van der Miesen et al., 2018a, Zucker et al., 2017) cited the same study by Vanderlaan et al. (2015). In this study, Vanderlaan et al. (2015) found that high birth weight was associated with high autistic traits and high gender nonconformity.

#### Extreme Male Brain

The extreme male brain (EMB) theory states that autistic people have an extreme male brain type (Baron-Cohen, 2002). Within this theoretical framework, a “male” brain type applies to those who are significantly better in systemizing than empathizing and a “female” brain type to those with the opposite cognitive profile. Baron-Cohen (2002) defines empathizing as the drive to identify and respond to others’ emotions or thoughts and systemizing as the drive to analyze and derive variables or underlying rules. The EMB theory is ﻿an extension of the empathizing-systemizing (ES) theory, which it implies that autistic people have diminished empathizing and increased systemizing abilities (Baron-Cohen, 2009). The EMB theory may be interpreted in such a way that it explains both ASC and TGM in people assigned female at birth (AFABs): AFABs with a more “male” brain would experience more ASC traits as well as more masculinity. Following this reasoning, most articles supporting the EMB theory propose it as an explanation for trans autistic AFABs only, while offering other explanations for trans autistic people assigned male at birth (AMABs). The EMB theory was mentioned in 11 of the included papers. Four of those claimed evidence for and three claimed evidence against the theory. Nobili et al. (2018) stated that their results potentially support the EMB theory based on two findings: trans AFABs more often scored in the clinical range on the Autism Quotient-Short (AQ-Short) compared to cis AFABs and trans AFABs more often scored in the clinical range on the AQ-Short compared to trans AMABs. In a second study, Nobili et al. (2020) subscribed to the EMB theory again, as they replicated the previous finding with respect to a difference between AFABs and AMABs on the AQ-Short. Kung (2020) similarly found results consistent with the EMB theory: they reported elevated autistic traits and systemizing in trans AFABs compared to cis AFABs, but they reported no difference between cis and trans AMABs. The fourth paper supporting the EMB theory was a study by Stagg and Vincent (2019)—however, they also claimed evidence against it. Stagg and Vincent (2019) found a higher rate of ASC traits and systemizing in trans adults compared to cis adults, which was primarily driven by AFABs. Still, trans AMABs showed higher rates compared to cis AMABs as well, and so Stagg and Vincent (2019) indicated a more nuanced theory than the EMB theory is needed. Two more articles claimed support against the EMB theory. Heylens et al. (2018) and van der Miesen et al. (2018a) both did not find a difference between AFABs and AMABs regarding ASC traits in trans people.

#### Prenatal Hormones

Closely related to the EMB theory is the idea that prenatal hormones contribute to the co-occurrence of ASC and TGM. According to this theory, androgen exposures to the developing fetal brain affect the development of both ASC and gender identity (i.e., an association between testosterone, masculinity, and ASC). Similar to the EMB theory, this theory predicts a higher incidence of TGM in autistic AFABs compared to autistic AMABs. Prenatal hormones were mentioned as an explanation in 16 papers, often together with the EMB theory. None of the authors referred to their own data when arguing for or against this theory. Still, several authors made their arguments based on other studies. For example, Zucker et al. (2017) argued for the role of prenatal hormones by referring to Vanderlaan et al. (2015), who suggested that risk factors for ASC were associated with lower prenatal testosterone levels in trans AMABs. In contrast with this, George and Stokes (2018) cited Auyeung et al. (2009), who found that elevated levels of fetal androgens were associated with ASC traits. Nobili et al. (2020) discussed the effects of androgen exposures at a later age; they found that a greater proportion of trans AFABs showed ASC caseness after gender-affirming hormone treatment than before treatment. Conversely, Heylens et al. (2018) argued against a role for prenatal hormones based on a study by Kung et al. (2016), who could not find an association between prenatal androgen exposure and autistic traits in children. Again, it is noteworthy that most studies presented prenatal hormones as an explanation for AFABs only.

#### Genetic Factors

A fourth biological explanation was identified in genetic factors. According to this explanation, there is a common underlying genetic predisposition for ASC and TGM. Genetic factors were mentioned as an explanation four times without claimed empirical evidence. Two of the studies, Cheung et al. (2018) and Zucker et al. (2017), referred to other studies on a possible genetic link between ASC and TGM. Based on the study by Vanderlaan et al. (2015), Cheung et al. (2018) proposed shared (epi)genetic factors for gender identity and ASC. Correspondingly, Zucker et al. (2017) referred to a study that found an association between the scores of mothers on the social responsiveness scale (SRS) and gender-variant behavior in their children (Shumer et al., 2015).

### Psychological Theories

#### Gender Development

This explanation proposes an atypical gender development as the reason for the high prevalence of TGM among autistic people. “Atypical” may entail a delayed gender development (e.g., Hisle-Gorman et al., 2019; Kallitsounaki et al., 2020) or differences in forming self-concept and/or self-categorization (e.g., Kallitsounaki & Williams, 2020b; Cooper et al., 2018). An evident direction is present in this theory, where ASC is the variable leading to TGM. Gender development was mentioned as an explanation seven times, and no evidence for or against it was offered in the included papers. For example, Kallitsounaki et al. (2020) proposed a role for gender development by citing Zucker et al. (1999), who found a developmental lag of gender constancy in GD-referred children. Kallitsounaki et al. (2020) argued that ASC may be associated with a delayed development of gender constancy, making autistic people more prone to TGM. Likewise, Cooper et al. (2018) suggested that differences in the ability to self-categorize in autistic people may lead them to develop more idiosyncratic gender identities compared to neurotypical people. Furthermore, a qualitative study by Hillier et al. (2019) described a participant who felt like their ASC had affected the development of their gender identity, rather than their understanding of gender identity (see “Rigidity” section and “Theory of Mind/Mentalizing” section for theories on understanding gender identity).

#### Obsessions

An obsession is an idea or thought that continually preoccupies or intrudes on a person’s mind (Oxford Dictionary n.d.). Regarding the role of obsessions in the ASC-TGM link, there were two interpretations present in the included papers: obsessions were interpreted as a manifestation of TGM leading to specific autistic traits (i.e., restricted or repetitive interests) or as an expression of ASC leading to (temporary) TGM. In total, obsessions were mentioned 14 times throughout the 36 papers. One paper offered evidence for and four papers offered evidence against obsessions as an explanation. Zucker et al. (2017) claimed evidence for obsessions based on their finding that GD-referred children had elevated rates of obsessions compared to referred and non-referred controls. They considered obsessions an established ASC trait and argued that ASC leads to TGM rather than the other way around. Conversely, Strang et al. (2018b) concluded that their qualitative data stand in contrast with the idea that TGM in autistic people is driven by superficial obsessional gender interests. In another study by Strang et al. (2018a), obsessions were not included in the clinical guidelines for co-occurring ASC and TGM, because 36% of their expert panel had not experienced this phenomenon in practice. In line with this, van der Miesen et al. (2018b) concluded their data did not fully correspond with the idea that intense gender interests in autistic people lead to TGM. In a study on GD-referred youth, van der Miesen et al. (2018a) argued that the ASC-TGM link cannot be attributed to any one subdomain of ASC—including intense interests—as they found that all ASC subdomains were elevated. Walsh et al. (2018) similarly did not find a specific elevation of ASC subdomains associated with intense interests in trans autistic people compared to cis autistic people.

#### Rigidity

Rigidity may be defined as the tendency to form and perseverate in the use of specific mental or behavioral sets (Schultz & Searleman, 2002). It has been considered a characteristic of ASC, as ASC has been found to be associated with reduced cognitive flexibility (D’Cruz et al., 2013). Regarding the ASC-TGM link, the rigidity theory entails that cognitive inflexibility due to ASC causes more rigid and concrete thinking about gender (i.e., more black-and-white views on what it means to be male/female), making autistic people more prone to TGM. Throughout the 36 papers, rigidity was mentioned as an explanation for the ASC-TGM link 13 times, often together with obsessions. There were no papers claiming evidence for and three papers claiming evidence against rigidity. Walsh et al. (2018), van der Miesen et al. (2018a, b) provided the same evidence against rigidity as they did against obsessions: none of them found an association between TGM and ASC traits associated with rigidity (i.e., difficulty with change or routine and switching subdomains).

#### Sensory Processing

Sensory processing is related to obsessions as an explanation for the ASC-TGM link (see “Obsessions” section). According to this theory, preferences for or over/under-responsivities to specific kinds of sensory stimuli are a shared factor in ASC and TGM. Specifically, it proposes that a need for certain sensory input may explain gender-related obsessions. Sensory processing was mentioned in two papers that both claimed evidence for it. First, van der Miesen et al. (2018a) proposed different underlying mechanisms for the ASC-TGM link between AMABs and AFABs, as they found that trans AMABs scored higher than trans AFABs on the “stereotyped” subdomain of the Children’s Social Behavior Questionnaire (CSBQ). The authors interpreted this finding as sensory processing being the explanation for gender-related interests in trans AMABs (e.g., a preference for glitter and soft clothes). Nabbijohn et al. (2019) also found an association between TGM and the “stereotyped” subdomain on the CSBQ, and stated that their finding was consistent with the hypothesis that intense gender interests are related to preferences for specific kinds of sensory input. Additionally, they found an association between TGM and sensory processing disorder (SPD). As there is some overlap between the symptomology of ASC and SPD, Nabbijohn et al. (2019) speculated this latter finding may be an extension of the ASC-TGM link.

#### Sexual Orientation

The role of sexual orientation in the co-occurrence of ASC and TGM was discussed three times throughout the included studies, without claims of evidence for or against it. The interpretation of the role of sexual orientation in the ASC-TGM link differed per study. One study by George and Stokes (2018) found that GD traits mediated the relationship between ASC traits and sexual orientation. In this paper, the authors mentioned a possible relationship between sexual orientation and gender experience in some autistic people and suggested that this relationship is in turn associated with the strength of ASC traits. Another study by Strang et al. (2018a) looked at the experiences of clinical experts. The authors found that a majority of these experts reported that some gay/bisexual autistic people assume they are a different gender because of their sexual orientation. A third study by Vermaat et al. (2018) found an association between ASC traits and sexual attraction to those of another gender than their birth-assigned sex in trans AFABs, but not in trans AMABs. Vermaat et al. (2018) interpreted this as an indication that screening for ASC traits is especially useful for trans AFABs who are attracted to those of another gender than their birth-assigned sex (Vermaat et al., 2018).

#### Theory of Mind/Mentalizing

Theory of mind (ToM) can be defined as the ability to attribute mental states, such as desires, emotions, beliefs, or intents, to oneself and others (Baron-Cohen et al., 1985). Regarding the ASC-TGM link, this explanation hypothesizes that deficits in ToM/mentalizing abilities due to ASC lead to TGM. Kallitsounaki and Williams (2020b) specified this by suggesting that autistic people, due to reduced ToM/mentalizing abilities, do not internalize stereotypical attributes of their birth-assigned sex. There were seven mentions of this theory, with three papers claiming support based on empirical data and no papers claiming evidence against it. Kung (2020) found significantly lower scores on the Reading the Mind in the Eyes (RMIE) test in trans AFABs compared to normative data. According to Kung (2020), their findings suggest a reduced ToM in trans AFABs, which may be interpreted as support for the mind-blindness theory (i.e., autistic children having a delayed development of ToM; Baron-Cohen, 2009). Kallitsounaki and Williams (2020b) proposed mentalizing as a contributing factor to the co-occurrence of ASC and TGM based on their finding that scores on the RMIE test moderated the relation between ASC traits and TGM. In a second study, Kallitsounaki et al. (2020) found that mentalizing mediated the ASC-TGM link.

#### Weakened Sex Differences

According to this explanation, a high prevalence of TGM in autistic people is due to weakened sex differences (i.e., autistic men being less masculine/more feminine and autistic women being less feminine/more masculine). Weakened sex differences were mentioned as an explanation four times, with two papers claiming support for it and no papers claiming evidence against it. Vermaat et al. (2018) found that trans AFABs had similar AQ scores to neurotypical males and that trans AMABs had similar AQ scores to neurotypical females. According to Vermaat et al. (2018), these findings underscore weakened sex differences in ASC. Vermaat et al. (2018) and van der Miesen et al. (2018a) both cited Beacher et al. (2012), who found less sex differences in the brain structure of autistic people compared to neurotypical people. The second paper supporting the theory of weakened sex differences was the study by Kallitsounaki and Williams (2020a). Based on their finding that ASC traits were associated with lower conscious identification with masculine/feminine attributes, Kallitsounaki and Williams (2020a) proposed a link between ASC and diminished gender-differentiated identity/self-concept.

### Social Theories

#### Feeling Different

The term “feeling different” here refers to the internal experience of feeling different from others. Concerning the co-occurrence of ASC and TGM, there were 10 mentions of feeling different as an explanation and no claims of empirical evidence. How feeling different may play a role in the ASC-TGM link has been interpreted in different ways. For example, Fielding and Bass (2018) suggested that TGM in autistic people is related to feeling different in general. Furthermore, Cooper et al. (2018) proposed that TGM in autistic people is associated with a lower sense of affiliation with gender groups compared to neurotypical people. Alternatively, different studies proposed that ASC traits in trans people reflect feelings of not fitting in due to TGM rather than actual ASC symptomology (Warrier et al., 2020; Akgül et al., 2018).

#### Minority Stress

Related to the theory on feeling different is the theory on minority stress. Minority stress may be defined as the stress experienced from external events due to being a minority (e.g., being bullied, left out, or ignored). It was named as a factor in the ASC-TGM link 18 times, with no claims of evidence for and one claim of evidence against it. Generally, the theory on minority stress was interpreted as TGM leading to (seemingly) autistic traits. For instance, various papers discussed how autistic traits in trans people possibly do not reflect actual ASC caseness, but rather social difficulties and non-supportive environments due to TGM (e.g., Cheung et al., 2018; Nobili et al., 2018; Leef et al., 2019; Russell et al., 2020). Stagg and Vincent (2019) and van der Miesen et al. (2018a) noted that even though minority stress could explain the elevation of social difficulties in trans people, it could not explain the elevation in other subdomains of ASC. Nobili et al. (2020) stated that minority stress cannot explain the ASC-TGM link, based on their finding that scores on the AQ-Short remained stable in trans adults before and after gender-affirming treatment. According to the authors, their findings reflect stable ASC traits rather than state responses to the social challenges due to TGM. Several qualitative studies discussed how minority stress may be experienced as a “double whammy,” as trans autistic people are marginalized on two fronts (Coleman-Smith et al., 2020; Hillier et al., 2019; Kuvalanka et al., 2018).

#### Resistance to Social Norms

This theory proposes that TGM is more common amongst autistic people because they are less susceptible to societal prejudice/pressure than neurotypical people. In other words, this explanation entails that autistic people are free from normative influences when forming their gender identity, whereas neurotypical people are influenced by the gender binary norm. There were 18 mentions of this theory in the included papers. One study claimed evidence for, and no studies claimed evidence against the explanation. In an ASC population, Walsh et al. (2018) found that 15% of the participants were trans and that only 6% of these trans participants identified as binary. According to the authors, the elevated nonbinary identities among trans autistic people are consistent with hypotheses on autistic resistance to social conditioning. Subsequently, Walsh et al. (2018) argued that resistance to social norms may promote both the initial self-recognition and subsequent disclosure of trans identities in autistic people. May et al. (2017), Kallitsounaki and Williams (2020a), and Kallitsounaki et al. (2020) suggested that problems with representing others’ perspectives (see “Theory of Mind/Mentalizing” section) may facilitate autistic people to come out as trans without concern for societal prejudice.

#### Social Communication

The last identified factor in the co-occurrence of ASC and TGM is social communication. This theory was mentioned 9 times, with one paper claiming evidence for it and one paper claiming evidence against it. Slightly different interpretations were given on how social communication relates to the ASC-TGM link. For instance, Cooper et al. (2018) suggested that difficulties in social communication in autistic people result in little understanding of gender norms, while Akgül et al. (2018) implied that difficulties in social communication could be a common phenomenon between ASC and TGM. Van der Miesen et al. (2018a) claimed evidence for social communication as an explanation for the ASC-TGM link. According to the authors, their findings (i.e., high scores on the social subdomain of the CSBQ in GD-referred children) point to social communication difficulties being involved in the association between ASC and TGM. Contrary to van der Miesen et al. (2018a), Nabbijohn et al. (2019) found that the tendency to seek out and engage in social interactions was not associated with TGM in the general population. According to Nabbijohn et al. (2019), their findings raise doubts about social communication being the basis of the ASC-TGM link.

## Discussion

This review provided the first comprehensive overview of the existing theories on the co-occurrence of ASC and TGM and the available evidence for or against them since 2016. When looking at the results at first glance, two things stand out: less than half of the included papers provided explanations for the ASC-TGM link based on their own data (15 out of 36 papers) and over a third of the mentioned theories lacked any claim of evidence for or against them (6 out of 15 theories). In short, the current findings demonstrate that the empirical basis for all theories on the ASC-TGM link is limited. This may be interpreted within the limitations of the available literature and theories. On the one hand, the literature is limited in the sense that many studies only report the prevalence of the co-occurrence of ASC and TGM without researching the possible explanatory factors for the ASC-TGM link. To be able to provide the best care for trans autistic people, it is necessary to consider not only the existence but also the implications of the ASC-TGM link. On the other hand, some theories are difficult to (dis)prove: certain conceptual social or psychological variables may be less measurable than concrete psychological or biological variables. Critical appraisal of theoretical explanations for the ASC-TGM link is important due to the role of science in informing debates around policy in transgender healthcare and the extant use of the co-occurrence as a barrier to autistic people accessing that care. Below, the findings are evaluated in more depth, and implications are discussed.

### Biological Explanations

There are some noteworthy observations when looking closer at the biological explanations for the ASC-TGM link. One such observation is that no empirical evidence was provided for or against three out of four biological explanations (birth weight, genetic factors, and prenatal hormones). Moreover, all articles that mentioned birth weight as an explanation for the ASC-TGM link referred to one and the same study (Vanderlaan et al., 2015). As such, it seems unlikely that birthweight could explain the ASC-TGM link, especially since high birthweight is not considered an established factor for ASC (Gardener et al., 2011). The theory on genetic factors similarly had little empirical underpinning, with the included articles referencing two previous studies only (Vanderlaan et al., 2015; Shumer et al., 2015). The one biological theory that received claims of evidence was the EMB theory. Based on the evidence (i.e., 4 claims for and 3 against), the EMB theory could possibly be considered an explanatory factor for the ASC-TGM link in AFABs. However, it cannot be the sole explanatory factor as it does not account for the co-occurrence of ASC and TGM in AMABs. The EMB theory additionally does not explain why there would be a sharp division in the mechanisms behind TGM between autistic AMABs and autistic AFABs. Consequently, the EMB theory may be considered an unlikely explanation for the ASC-TGM link.

### Psychological Explanations

Several remarks can be made with respect to the psychological explanations for the ASC-TGM link. First, there were no claims of evidence for or against the theories on gender development and sexual orientation. Although this lack of empirical data could reflect the difficulties of measuring psychological constructs, it is also possible that these theories are not researched because they are simply unpopular. The theory on sexual orientation for instance is rather outdated, considering the currently available research and awareness on the topics of sexuality, gender modality, and ASC (Øien et al., 2018). Secondly, the theories on obsessions and rigidity mostly or solely received claims of evidence against them. Based on this, it may be concluded that neither obsessions nor rigidity are likely to play a role in the ASD-TGM link. Thirdly, it is noticeable that the theory of sensory processing has as many evidence claims as mentioned (i.e., two; van der Miesen et al., 2018a; Nabbijohn et al., 2019). Although both claims are in favor of the theory, there are two problems with this theory: sensory processing is not an established trait of TGM or GD (APA, 2013), and van der Miesen et al. (2018a) provide the theory as an explanation for AMABs only. Therefore, there is no strong basis for the role of sensory processing in the ASC-TGM link despite the claimed evidence. The fourth point of discussion is provided by an assumption made in the theory on ToM/mentalizing—namely that ToM is impaired in autistic people. Although evidence for this theory is claimed three times, impaired ToM in autistic people has been challenged (Milton, 2012; Gernsbacher & Yergeau, 2019). If ToM is not necessarily impaired in autistic people, an explanatory role for ToM in the ASC-TGM link also seems questionable. Lastly, the theory of weakened sex differences received two claims of evidence for it. This theory could possibly play a role in the explanation of the ASC-TGM link, as it provides an explanation for both AMABs and AFABs, and the evidence was claimed based on different measurements (Kallitsounaki & Williams, 2020a; Vermaat et al., 2018).

### Social Explanations

There were very few claims of evidence for or against the social theories. While they received the most mentions of all three categories (55 out of the 140 total mentions), the social theories only received 4 out of the 26 evidence claims. As previously mentioned, this may be explained by the difficulties with measuring social constructs. A point of discussion regarding the social explanations is the distinction that has been made between the theories on feeling different and minority stress. One could argue that these are too alike to be distinct theories. While recognizing the interplay between feeling different and minority stress, the decision was made to separate them based on a key difference. While feeling different was defined as the internal experience of a person, minority stress was defined as the stress inflicted by a person’s external environment. The theories on feeling different and minority stress were mentioned in 10 and 18 of the included papers, respectively. They were mentioned together in seven papers. This demonstrates that they are, at least to some extent, distinct theories. Looking at the theory on social communication, it stands out that this theory received contradicting claims of evidence. Based on their results on the CSBQ, van der Miesen et al. (2018a) claimed evidence for the theory, while Nabbijohn et al. (2019) claimed evidence against it. As such, there is no substantial empirical basis for or against the role of social communication in the ASC-TGM link. The fourth and last social explanation, the theory of resistance to social norms, could possibly be an explanatory factor for the ASC-TGM link. This theory converges with a theoretical account of autism drawing on Bayesian decision theory. The Bayesian decision theory suggests that autistic people have “flattened priors,” where a “prior” is defined as the probability distribution assigned to different possibilities before new information is interpreted (Jackson-Perry, 2020; Pellicano & Burr, 2012; Walsh et al., 2018). This would entail that autistic people, compared to neurotypical people, give past experience and knowledge less weight when interpreting new information. In other words, autistic people would be less “confident” in the validity of (unconscious) generalization from past experiences to new situations. In the context of the theory of resistance to social norms, this implies that autistic people can more readily update their perceptions when past experience (e.g. social conditioning) hinders understanding of new information (e.g. other gender modalities).

### Contradiction and Complementarity

When looking at all theories, there are potentially contradictory and complementary explanations to be found. For instance, the theories on minority stress and resistance to social norms are seemingly contradictory: while the theory on minority stress suggests that autistic people are marginalized and affected by social ostracism, the theory on resistance to social norms suggests that autistic people do not conform to social norms. However, these theories are not necessarily mutually exclusive: the fact that a person does not internalize social norms as their own does not mean that they are insensitive to negative reactions of their surroundings. A more substantial contradiction may be found between the theories on rigidity and sexual orientation, and the theories on weakened sex differences and resistance to social norms. While the former theories imply that autistic people have inflexible ideas about gender, the latter theories imply autistic people are less constrained by the gender-binary norm. This is not surprising, as the findings of the current review suggest that rigidity and sexual orientation are less likely explanatory factors in the ASC-TGM link than weakened sex differences or resistance to social norms. Conversely, there are several theories that may be interpreted as complementary. For brevity, two of the less obvious combinations are discussed here. Firstly, the theory on weakened sex differences may be complementary to the theory on feeling different. Cooper et al. (2018) found that autistic people have lower gender identification than neurotypical people. When someone does not identify with their assigned gender group, they may experience feeling different from others (who do identify with a gender group), causing them to also experience weakened socially conditioned sex differences. Secondly, the EMB-theory of ASC seeks to explain ASC in terms of empathizing/systemizing, which may be considered complementary to the theory on weakened sex differences. It has been suggested that the binary gender system is “imperfect” (i.e., no one-size-fits-all rules for male/female behavior and changing gender perception according to social trends; Kristensen & Broome, 2015). Consequently, people with high systemizing abilities would be likely to challenge such an imperfect system, leading them to discover alternative nonbinary models for gender. This line of thought corresponds with Baron-Cohen’s (2002) definition of systemizing in the EMB theory (i.e., the drive to analyze and derive variables or underlying rules) as well as with the notion that autistic people are less affiliated with either masculinity or femininity.

Another observation that stands out when looking at all theories, is the difference in reasoning between certain theories. Some theories reason from the deficit model for ASC (e.g., the theories on rigidity, ToM/mentalizing, and social communication) whereas other theories reason from the neurodiversity model for ASC (e.g., the theories on weakened sex differences and resistance to social norms). There are two reasons to argue that theories based on a neurodiversity model might explain the ASC-TGM link better than that reasoning from a deficit model. First, the neurodiversity model lends itself better for reasoning along both sides of the spectrum: this model may account for the co-occurrence of ASC and TGM as well as for the co-occurrence of neurotypicality and cisgender modalities (Walsh et al., 2018). Second, unlike the deficit model, the neurodiversity model does not have the drawbacks of potential social harm or impeded scientific understanding due to the pathologizing of ASC (Dinishak, 2016).

### Methodological Explanations

As this review focused on the question “why is there a link?” rather than “is there a link?”, the presence of the ASC-TGM link was assumed based on the available literature (see Thrower et al., 2020). For this reason, only theoretical explanations, likewise assuming the presence of the link were discussed. However, several papers provided methodological explanations as well. The three most mentioned methodological explanations were the non-specificity effect, difficulties with diagnosing autistic women, and biases. The non-specificity effect entails that ASC and/or TGM are linked with co-occurring diagnoses in general. Some of the included studies controlled for the non-specificity effect by comparing their results to a referred control group. For example, Zucker et al. (2017) and Leef et al. (2019) found partial specificity for ASC traits in GD-referred children compared to referred controls, and May et al. (2017) found no specificity for gender incongruence in autistic youth compared to referred controls. The second mentioned methodological explanation refers to how the underdiagnosing of autistic women may affect the ASC-TGM link (Dworzynski et al., 2012; Robinson et al., 2013). An example of this is presented by Hisle-Gorman et al. (2019), who found that TGM was more prevalent in AFABs than in AMABs for neurotypical children but not for autistic children. Although they mention a genetic or biological link to explain their findings, they suggest it is most likely due to difficulties in diagnosing ASC in AFABs. The last methodological explanation concerns biases. For instance, Kallitsounaki and Williams (2020b) discuss the possibility of a selection bias due to opportunity sampling. Additionally, Russell et al. (2020) suggest that their parent-reported results may have been biased by parents viewing their child through a “gendered lens.” Concerning the methods, some general remarks can be made. Throughout the 36 studies, questionnaires such as the AQ and the SRS were often used to measure ASC. These methods are screening tools rather than diagnostic tools and only measure traits. As such, they cannot be used to assess actual ASC. Additionally, psychometrical issues have been found with the total AQ (-Short) scores (English et al., 2020) and the AQ-10 (Taylor et al., 2020). It is important to take these methodological limitations into account when interpreting the currently available research and when designing new research protocols involving ASC and/or TGM.

### Unlikely and Promising Explanations

After reviewing the theories, it seems unlikely just one theory may explain the ASC-TGM link in all trans autistic individuals. Instead, it seems most plausible that different theories are applicable to different people in different situations. For instance, the underlying mechanism for the ASC-TGM link may differ between people experiencing ASC more as a “barrier” (in line with the theories such as the theory on social communication) and people experiencing ASC more as a “protector” (in line with theories such as the theory on resistance to social norms). Although none of the identified theories on the ASC-TGM link had an established empirical basis, some theories are more promising than others. From the evaluation of the strengths and weaknesses of the claimed evidence per theory, it may be concluded that the theories on birthweight, sexual orientation, rigidity, and obsessions are unlikely explanations for the ASC-TGM link. Conversely, the theories on weakened sex differences and resistance to social norms could prove promising explanations. However, to make any convincing conclusions on which theories contribute to the ASC-TGM link more empirical evidence is needed.

### Future Directions and Implications

Several possibilities for future directions may be uncovered from the current overview. On the one hand, future research could explore promising theories to strengthen their empirical basis, such as those on weakened sex differences and resistance to social norms. On the other hand, theories that were neither likely nor unlikely could be studied to (dis)prove them based on empirical data, such as those on social communication, gender development, or feeling different. As there were only two included studies with longitudinal data (Nobili et al., 2020; Russell et al., 2020), longitudinal studies on the ASC-TGM link also present an important future direction. This type of study might prove relevant for theories that imply temporality of the ASC-TGM link (e.g., theories on obsessions or minority stress). Any further investigation of the ASC-TGM link should take the importance of matched and referred control groups into account, as well as the implications of the used measurements (e.g., screening vs. diagnostic tools). Most importantly, future research on the ASC-TGM link should not be limited to investigating the existence of such a link but should broaden its focus to understanding the consequences of the link for efforts to meet the needs of trans autistic people. The present review contributed to the growing body of research on the topic of the ASC-TGM link by providing an overview of all theories and their evidence on the co-occurrence of ASC and TGM since 2016, helping further understanding and treatment of trans autistic people in the future.
